# A georeferenced database of the edaphic biota currently available for Argentina

**DOI:** 10.3897/BDJ.11.e113079

**Published:** 2023-10-25

**Authors:** María C.V. Sanabria, Víctor N. Velazco, Gabriel Tolosa, Liliana B. Falco, Carlos E. Coviella

**Affiliations:** 1 Instituto de Ecología y Desarrollo Sustentable (INEDES) - Dept. of Basic Sciences, Universidad Nacional de Luján, Luján, Argentina Instituto de Ecología y Desarrollo Sustentable (INEDES) - Dept. of Basic Sciences, Universidad Nacional de Luján Luján Argentina; 2 Universidad Nacional de Luján, Luján, Buenos Aires, Argentina Universidad Nacional de Luján Luján, Buenos Aires Argentina

**Keywords:** occurrence, mites, springtails, earthworms, Oligochaeta, georeferencing, soil biota, ecosystem services, metacommunity, land use, soil biogeography

## Abstract

**Background:**

Soils have been studied and classified in terms of their physical and chemical characteristics, while the knowledge about biodiversity and the ecosystem processes that they support is lagging behind. Furthermore, the advance in scientific knowledge contributed by different researchers is dispersed and it is necessary to collect it to bring the big picture into focus. Today, it is possible to have the findings and data collected by different researchers, compile them and, based on technological advances, have tools that allow the information to be analysed in its entirety. The main objective of this work is to compile and systematise all the bibliographic information available on the main organisms that make up biodiversity in the soil: Acari, Collembola and Crassiclitellata in Argentina. This information will then allow us to link the composition and structure of the soil community with processes and flows in the ecosystem, and to estimate them at different scales and in soils with different anthropic impact. The database presented here gathers presence information on the mentioned taxa, their geographical location for the entire country, while preserving the identity and authorship of each scientific work retrieved. The taxonomic range of the organisms of the edaphic biota collected in this database ranges from class to subspecies and are registered, based on the taxonomic level reported by the original author in their research. The publications were obtained from Google Scholar, Scopus and JSTOR. In addition, records were added from INEDES theses, library searches, information requested from authors cited in other articles and unpublished works. In total, information was collected from 224 scientific publications, as well as personal information requested directly from some authors. The total number of registered individuals so far is 4838 of which 3049 specimens correspond to Acari, 944 to Classiclitellata and 845 belong to Collembola.

**New information:**

This work is the first to gather, in a single publication, the entire dataset for all the Acari, Collembola and Clitellata recorded for Argentina.

## Introduction

Our human activities, such as housing, health, clothing and food, are sustained by the use of natural resources. This use produces modifications in the environment that is the habitat of various biological communities and, at the same time, are an integral part of the ecosystems and the functions that occur therein. The study of biodiversity is presented as a challenge today because of the urgent need to know what types of organisms are present, where they are found and how biodiversity determines ecosystem functions, but, more importantly, because it is essential to maintain the structure of the edaphic ecosystem in the face of the different uses it is subjected to and to understand the close relationship with the edaphic processes that provide key ecosystem services and benefit human beings (Marichal et al. 2004, Phillips et al. 2019).

Integrated indices that group different indicators are used in a wide variety of disciplines because they cover complex and multidimensional concepts, synthesising a large amount of information in a simple and practical form. Currently, the evaluation of water quality shows extensive development in the use of different integrated indices, such as those used by the European Water Framework Directive (Chave 2001) or the Clean Water Act, the Federal Water Control Act, Water Pollution of the United States (ACT 2002). Constructing indices from biological data potentially constitutes a key tool to promote the care and rational sustainable use of soils. However, at present, there are no well-developed indicators for terrestrial systems (Knoepp et al. 2000) applicable at the regional level. This is why it is necessary to unify criteria, compile and synthesise existing information to achieve efficient use of resources that result in planning and correct regulation of sustainable land use. This systematised information will also be a valuable source of information to promote awareness and the adoption of protective measures (Rutgers et al. 2016).

**The importance of compiling soil fauna biodiversity data**

The systematic and permanent evaluation of the components that make up the state of the soil resource is an activity that requires indices and indicators that integrate and standardise complex and multidimensional information. This information must also be applicable at different scales to allow the understanding of the effects of the use of the soil resource and avoid its deterioration (Alkorta et al. 2003). There are currently standardised physical and chemical analysis techniques that evaluate the instantaneous state of the soil, but they do not evaluate the dynamic processes that affect structural stability and nutrient cycling that depend on the presence of biological activity in soils. It is necessary to evaluate the impact that different land uses can have on the organisms that compose the soil biota and, in this way, to generate biological indices and indicators that synthesise and account for the phenomena that occur in the soil.

Soil arthropods present a vast number of particularities that define them as efficient indicators of the functioning of the edaphic ecosystem. Amongst them are their great diversity, their ability to occupy microhabitats, their requirements for specific niches and their contribution to ecological cycles. In addition, they are highly sensitive to changes in environmental conditions and disturbances. They have a wide response capacity related to characteristics such as: body size, growth rates, dispersal capacity, adaptations to microclimatic conditions, their short reproductive cycles and their importance in food chains, in the degradation of the organic matter and flow of nutrients and energy in the system (Herrera and Cuevas 2003, Sanabria 2020).

The invertebrates present in the soil biota are a primary link in the physical and chemical dynamics of the soil. They directly influence the formation of biogenic structures, the cycling of nutrients, the formation of aggregates and the decomposition of organic matter, soil porosity and water retention capacity (Sanabria 2020). In this work, mites (Arachnida, Acari), springtails (Entognatha, Collembola) and earthworms (Oligochaeta, Crasiclitellata) are considered. Both Acari and Collembola have characteristics that make them excellent biological indicators and this criterion is accompanied by a number quantity of bibliographies and recent studies that address the topic (Bedano 2007, Socarrás and Izquierdo 2014). In the case of Clitellata, in addition to being considered good bioindicators, they are considered ecosystem engineers due to the structural processes that their activity produces in the soil.

Today, it is possible to systematise and organise a large amount of information distributed in a wide variety of formats. Through technological advances that allow the management of large amounts of data, it is possible to relate them to multiple factors linked to the different systems of land use and their effects on the soil ecosystem. At the moment, there is no work for Argentina that collects all the available information on the biodiversity of the country's soil biota in a single place. The construction of such a georeferenced database on Acari, Collembola and Crassiclitellata constitutes the first step to knowing the biodiversity currently recognised for Argentina and has been recently compiled by Sanabria et al. (2023).

## Project description

### Title

A Georeferenced Database of the Edaphic Biota Currently Available for Argentina

### Personnel

Maria Cynthia Valeria Sanabria, Víctor Nicolás Velazco, Gabriel Tolosa, Liliana B. Falco, Carlos E. Coviella, Anabela Plos

### Study area description

The sites where the relevant taxa were found are in the Neotropical Region, on the continent of South America, specifically in the Argentine Republic. Its extension is 13,761,274 km^2^ including the terrestrial areas, whose sovereignty is claimed by Argentina. The country has a wide surface coverage; therefore, it also has important climatic diversity, ranging from the tropical climates of the Chaco, Tucumán-Oranense and Misiones ecoregions, to the cold and dry climate of Patagonia.

### Design description

The database was built in two stages. In the first, bibliographic information on the taxa Acari, Collembola and Crassiclitellata was collected. The search for scientific works was carried out in different online search sites and physical documents of researchers from INEDES and libraries. In the second stage, the data were was integrated into the database respecting taxonomic levels and authorship of the initial researcher. The working database that compiles all the gathered information was designed following the best practices of relational database design to allow the efficient representation of data. It also enables querying the database in a flexible way.

### Funding

This project has been funded by a Doctoral Scholarship to María Cynthia Valeria Sanabria from the Concejo Nacional de Investigaciones Científicas (CONICET-Argentina), through the research programme in Terrestrial Ecology of Universidad Nacional de Luján and with the support of the Instituto de Ecología y Desarrollo Sustentable (UNLu-CONICET). There was also logistical support from the GBIF Argentina node, which is in charge of standards control, review and hosting of data and metadata.

## Sampling methods

### Study extent

The study area covers the entire territory of the Argentine Republic. Bibliographic works with information on the taxa Acari, Collembolla and Clitellata were collected from different online repositories. The first recorded work is from 1902 and the last one is from 2023.

### Sampling description

Database building:

A database was built containing all the information available for Argentina on Acari, Collembola and Crassiclitellata taxa. The building of the data base was carried out in two stages as described below.

### Step description

Step one: Data collection.

A comprehensive search was performed on the taxa of Acari, Collembola and Crassiclitellata for works carried out in all of Argentina, from since as far back in time as possible (Suppl. material 1, Velazco (2023)). The works include theses from INEDES, online searches from Google Scholar, Scopus and JSTOR, personal requests to authors mentioned in the bibliography, Universidad de Buenos Aires library and Argentina's National Library. In each search engine, it was necessary to use several query variations to obtain get a higher document recall.

In Scopus, the following strings were used: ALL((microarthropods OR springtails OR mites OR oribatida OR mesostigmata OR prostigmata OR astigmata) AND (argentina) AND soil AND (family OR genus OR species)) AND (LIMIT-TO (SUBJAREA, "AGRI") OR LIMIT-TO (SUBJAREA, "ENVI") OR LIMIT-TO (SUBJAREA, "MULT") OR LIMIT-TO (SUBJAREA, "EART")) AND (LIMIT-TO (EXACTKEYWORD, "Collembola") OR LIMIT-TO (EXACTKEYWORD, "Acari") OR LIMIT-TO (EXACTKEYWORD, "Soil Fauna")) AND (LIMIT-TO (LANGUAGE, "English") OR LIMIT-TO (LANGUAGE, "Spanish") OR LIMIT-TO (LANGUAGE, "Portuguese"));

ALL((microarthropods OR springtails OR mites OR oribatida OR mesostigmata OR prostigmata OR astigmata or earthworm) AND (argentina) AND soil AND (family OR genus OR species)) AND (LIMIT-TO (SUBJAREA, "AGRI") OR LIMIT-TO (SUBJAREA, "ENVI") OR LIMIT-TO (SUBJAREA, "MULT") OR LIMIT-TO (SUBJAREA, "EART")) AND (LIMIT-TO (EXACTKEYWORD, "Collembola") OR LIMIT-TO (EXACTKEYWORD, "Acari") OR LIMIT-TO (EXACTKEYWORD, "Soil Fauna")) OR LIMIT-TO (EXACTKEYWORD, "earthworm")) AND (LIMIT-TO (LANGUAGE, "English") OR LIMIT-TO (LANGUAGE, "Spanish") OR LIMIT-TO (LANGUAGE, "Portuguese")).

In Google Scholar, the initial search was for each taxon in Argentina; for example, "Acari Argentina". This search provided only few publications. Therefore, a search was implemented for each group in each province, such as “Acari Salta Argentina”, both in Spanish and English.

JSTOR database was used for searching older publications. Additionally, if some work mentioned in a publication could not be found online or in libraries, the author was contacted directly to ask for the data. This was also the way unpublished works were obtained.

The occurrence records were georeferenced, based on the information provided by each original work. In this way, the occurrences were geographically located according to different strategies: a) if the work reported the exact coordinates, these were taken, b) if the publication referenced the sites in an image, then these were interpolated using a GIS tool and this approximation was taken as valid, c) if the works did not present exact information on the geographical coordinates, they were geolocated to the closest locations using Google Maps or Google Earth, d) Always, where possible, the georeferenced locations requested from the authors of the original work were used.

Additionally, the biological occurrence of the different taxa found, as well as their geographical locations, were recorded in this database and we also ensured that the ownership of each scientific work was preserved, adding the corresponding author to the registry.

Step two: Data integration.

The synonymy used by each researcher to identify the original work was preserved, including the key and nomenclature used by the authors during the development of their research. However, the taxonomy and nomenclature have changed over the years. That is why current systematic listings were chosen for each group according to the current taxonomic structure.

In order to unify the nomenclature for Acari, the systematic lists of Subias (2022) and Pachl et al. (2020) in the suborder Oribatida were used for the Sarcoptiformes. For the infraorder Astigmata, as well as for the orders Mesostigmata and Trombidiformes, the list proposed in Krantz and Walter 2009 and Krantz and Walter (2009), Zhang (2011) was used.

For Collembola, we followed the criteria put forth by Deharveng (2004), Zhang (2011) and Bernaba Lavorde and Palacio-Vargas (2020), where for works that used a different taxonomic level or a type of classification that fell into disuse, the data were was incorporated into the database at the higher taxonomic level.

For grouping the order Crassiclitellata, the information collected by Brown and Fragoso (2007) and by James and Davidson (2012) was used. Additionally, we also followed the considerations by Schmelz et al. (2021) that proposes updating in Oligochaeta (Annelida, Clitellata) to order.

For all taxonomic groups, when a specimen was tagged with a question mark indicating an ID doubt (?) or with the abbreviations aff. or cf., it was registered at the next higher level (Lanteri 2000, Acosta 2007). For instance, the individual registered as Scheloribatesaff.bidactylus, is counted as of the genus *Scheloribates*.

## Geographic coverage

### Description

This work covers all of Argentina's geography, as the collection of the information was performed as described above, for the entire territory of the country.

**Figure 1.**
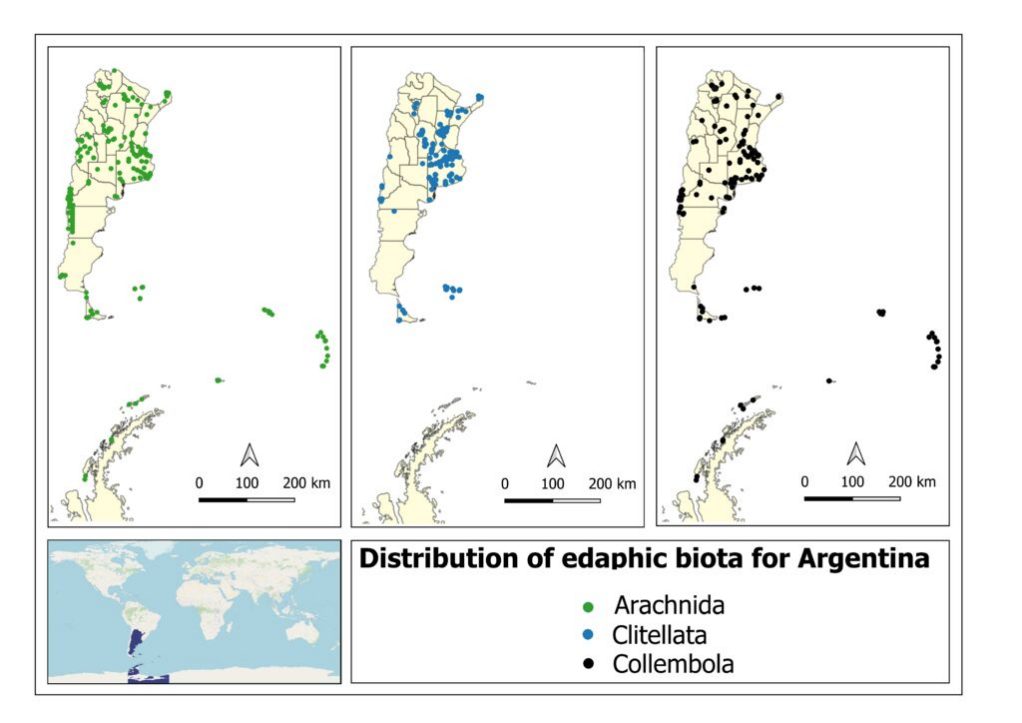
Site locations where specimens were collected in Argentina (Arachnida, Collembola and Clitellata).

Table 1 shows the description of the three principal classes working in the database (Fig. 1).

### Coordinates

-68.1 and -22.056 Latitude; -73.163 and -26.367 Longitude.

## Taxonomic coverage

### Description

This dataset of organisms of the edaphic biota in Argentina covers different taxonomic levels of the Clitellata (Oligochaeta), Collembola and Acari classes. It shows the Orders, Infraorders, Superfamilies and Families widely recognised in the cited bibliography.

Table 2 presents the summary of numbers of the order of edaphic taxa found in Argentina.

For full list of taxa, which includes 1086 different taxa, see Suppl. material 2.

### Taxa included

**Table:** 

Rank	Scientific Name	
class	Clitellata Michaelsen, 1919	
order	Crassiclitellata Jamieson, 1988	
superfamily	Megascolecoidea	
family	Acanthodrilidae Claus, 1880	
family	Megascolecidae Rosa, 1891	
family	Ocnerodrilidae Beddard, 1891	
superfamily	Lumbricoidea	
family	Lumbricidae Rafinesque-Schmaltz, 1815	
superfamily	Glossoscolecoidea	
family	Glossoscolecidae Michaelsen, 1900	
superfamily	Enchytraeoidea	
family	Enchytraeidae Vejdovsky, 1879	
class	Collembola Lubbock, 1870	
order	Entomobryomorpha Börner, 1913	
superfamily	Isotomoidea Schäffer, 1896	
family	Isotomidae Schäffer, 1896	
superfamily	Entomobryoidea Schäffer, 1896	
family	Entomobryidae Schäffer, 1896	
family	Paronellidae Börner	
family	Microfalculidae Massoud y Betsch, 1966	
superfamily	Tomoceroidea Szeptycki A, 1979	
family	Tomoceridae Schäffer, 1896	
order	Poduromorpha Börner, 1913	
superfamily	Hypogastruroidea Börner, 1906	
family	Hypogastruridae Börner, 1906	
superfamily	Neanuroidea Börner, 1901	
family	Neanuridae Börner, 1901	
family	Brachystomellidae Stach, 1949	
family	Odontellidae Massoud, 1967	
superfamily	Onychiuroidea Lubbock, 1867	
family	Onychiuridae Lubbock, 1867	
family	Tullbergiidae Bagnall, 1935	
superfamily	Poduroidea Latreille, 1804	
family	Poduridae Latreille, 1804	
superfamily	Isotogastruroidea Thibaud y Najt, 1992	
family	Isotogastruridae Thibaud y Najt, 1992	
order	Neelipleona Massoud, 1971	
family	Neelidae Folsom, 1896	
order	Symphypleona Börner, 1901	
superfamily	Katiannoidea Börner, 1913	
family	Katiannidae Börner, 1913	
family	Spinothecidae Delamare Deboutteville, 1961	
family	Arrhopalitidae Stach, 1956	
superfamily	Sminthuroidea Lubbock, 1862	
family	Sminthuridae Lubbock, 1862	
family	Bourletiellidae Börner, 1912	
superfamily	Sminthuridoidea Börner, 1906	
family	Sminthurididae Börner, 1906	
superfamily	Dicyrtomoidea Börner, 1906	
family	Dicyrtomidae Börner, 1906	
class	Arachnida Cuvier, 1812	
subclass	Acari Leach, 1817	
order	Trombidiformes Reuter, 1909	
suborder	Prostigmata Kramer, 1877	
superfamily	Paratydeoidea Baker, 1949	
family	Paratydeidae Baker, 1949	
superfamily	Anystoidea Oudemans, 1936	
family	Anystidae Oudemans, 1936	
superfamily	Trombidioidea Leach, 1815	
family	Trombidiidae Leach, 1815	
superfamily	Erythraeoidea Robineau-Desvoidy, 1828	
family	Erythraeidae Robineau-Desvoidy, 1828	
family	Smarididae Kramer, 1878	
superfamily	Eupodoidea Koch, 1842	
family	Rhagidiidae Oudemans, 1922	
family	Eupodidae Koch, 1842	
family	Penthalodidae Thor, 1933	
superfamily	Bdelloidea Dugès, 1834	
family	Cunaxidae Thor, 1902	
family	Bdellidae Dugès, 1834	
superfamily	Tydeoidea Kramer, 1877	
family	Tydeidae André, 1980	

## Temporal coverage

### Notes

The aim was to collect all the information available for the soil fauna of Argentina from since as far back as possible. The oldest cited work was published in 1902 and the newest was published in 2023.

## Usage licence

### Usage licence

Open Data Commons Attribution License

### IP rights notes

This work is licensed under a Creative Commons Attribution Non Commercial (CC-BY-NC 4.0) License.

## Data resources

### Data package title

A georeferenced database of the edaphic biota currently available for Argentina

### Resource link

https://doi.org/10.15468/4pcmjs

### Alternative identifiers

https://www.gbif.org/dataset/b863efea-ab18-47a6-bf8e-65fa8962a18a

### Number of data sets

1

### Data set 1.

#### Data set name

A georeferenced database of the edaphic biota currently available for Argentina

#### Data format

Darwin Core

#### Description

Soils have been studied and classified in terms of their physical and chemical characteristics, while the knowledge about biodiversity and the ecosystem processes that they support is lagging behind. Furthermore, the advance in scientific knowledge contributed by different researchers is dispersed and it is necessary to collect it to bring the big picture into focus.

Today, it is possible to have the findings and data collected by different researchers, compile them and, based on technological advances, have tools that allow the information to be analysed in its entirety. The main objective of this work is to compile and systematise all the bibliographic information available on the main organisms that make up biodiversity in the soil: Acari, Collembola and Crassiclitellata in Argentina. A second objective is to link the composition and structure of the soil community with processes and flows in the ecosystem and to estimate them at different scales and in soils with different anthropogenic impact.

The database presented here gathers presence information on the mentioned taxa, their geographical location for the entire country, while preserving the identity and authorship of each scientific work consulted. The taxonomic range of the organisms of the edaphic biota collected in this database ranges from class to subspecies and are registered, based on the taxonomic level reported by the original author in their research.

The publications were obtained from Google Scholar, Scopus and JSTOR. In addition, records were added from INEDES theses, library searches, information requested from authors cited in other articles and unpublished works. In total, information was collected from 224 published scientific works as well as personal information requested directly from some authors. The total number of registered individuals so far is 4838, of which 3049 specimens correspond to Acari, 944 to Classiclitellata and 845 belong to Collembola.

**Data set 1.** 

Column label	Column description
occurrenceID	A unique identifier for the occurrence, allowing the same occurrence to be recognised across dataset versions as well as through data downloads and use.
basisOfRecord	The individual record type, in this case, is cited material.
institutionCode	An identifier of the institution that has custody of the record.
collectionCode	An identifier of the dataset from which the record was derived.
catalogNumber	It is a unique identifier assigned to each taxon in the dataset.
higherClassification	A list (concatenated and separated) of taxon names that end in the range immediately above the referenced one. In this case kingdom, phylum and class.
kingdom	The full scientific name of the kingdom in which the taxon is classified.
phylum	The full scientific name of the phylum in which the taxon is classified.
class	The full scientific name of the class in which the taxon is classified.
order	The full scientific name of the order in which the taxon is classified.
family	The full scientific name of the family in which the taxon is classified.
genus	The full scientific name of the genus in which the taxon is classified.
subgenus	The full scientific name of the subgenus in which the taxon is classified.
specificEpithet	The name of the first or species epithet of the scientificName.
infraspecificEpithet	The name of the lowest or terminal infraspecific epithet of the scientificName.
scientificNameAuthorship	The authorship information for the scientificName.
scientificName	Reports the scientific name of the taxon.
taxonRank	Reports the taxonomic rank of the taxon.
lifeStage	Taxonomic rank
habitat	Reports in what type of environment the taxon was found.
year	Reports year of sampling.
higherGeography	A (concatenated and separate) list of geographical names that is less specific than the information captured in the locality term. In this case, they are continent, country and stateProvince.
continent	The name of the continent in which the occurrences are reported.
islandGroup	The life stage of the organism at the time the event was recorded.
island	The name of the island on which the taxa appears.
country	The name of the country in which the taxa appears.
countryCode	The standard code for the country in which the taxa occurs.
stateProvince	The name of the administrative region next smaller than the country in which the located taxa appear. In this case province.
locality	The specific description of the place.
decimalLatitude	The geographic latitude in decimal degrees, using the spatial reference system provided in geodeticDatum.
decimalLongitude	The geographic longitude in decimal degrees, using the spatial reference system provided in geodeticDatum.
geodeticDatum	The ellipsoid, geodetic datum or spatial reference system (SRS) on which the geographic coordinates given in decimalLatitude and decimalLongitude are based.
coordinateUncertaintyInMetres	The horizontal distance (in metres) from the given decimalLatitude and decimalLongitude that describe the smallest circle containing the georeferencing.
georeferencedBy	A list (concatenated and separated) of names of people who determined the georeference of the occurrences.
georeferencedDate	The year in which the occurrences were georeferenced.
georeferenceProtocol	A description or reference of the methods used to determine the spatial footprint, coordinates and uncertainties.
bibliographicCitation	A bibliographic reference for the resource.

## Additional information

Sanabria M C V, Velazco V N, Tolosa G, Falco L B, Coviella C E (2023). A georeferenced database of the edaphic biota currently available for Argentina. Version 1.12. Instituto de Ecologia y Desarrollo Sustentable (INEDES). Occurrence dataset https://doi.org/10.15468/4pcmjs accessed via GBIF.org on 2023-09-21.

## Supplementary Material

FB549C0D-1D1F-547D-B37F-9A4274AA7B0C 10.3897/BDJ.11.e113079.suppl1 Supplementary material 1 Bibliographic material used in the construction of the dataset Data type Bibliografic Brief description A bibliographic resource was built that contains all the information available on the organisms of the edaphic biota recorded from 1900 to the present day present in Argentina on the taxa Acari, Collembola and Crassiclitellata, soil fauna closely linked to the functioning of the ecosystem and ecosystem services. The construction of the database was carried out in two stages, namely, the collection of raw data and their integration afterwards.
This resource is also available on Zotero: https://www.zotero.org/groups/5184714/edaphic_biota_from_argentina File: oo_905942.bib https://binary.pensoft.net/file/905942 Sanabria, MCV

D080C20C-4B80-5ED9-BF9B-A2BE326BADD9 10.3897/BDJ.11.e113079.suppl2 Supplementary material 2 Full List Taxa of edaphobase from Argentina Data type List of taxa Brief description Complete list of taxa found, with their number of records, in the bibliography cited for the Argentine Republic. File: oo_905961.xlsx https://binary.pensoft.net/file/905961 Sanabria, MCV

## Figures and Tables

**Table 1.** Number of the principal classes of edaphic fauna registered in Argentina.

**State Province**	**Clitellata**	**Collembola**	**Arachnida**
Buenos Aires	463	384	1226
Chaco		2	69
Chubut	1	15	207
Corrientes	6	2	26
Córdoba	277	74	236
Entre Ríos	2	10	58
La Pampa	1	5	40
La Rioja		5	5
Mendoza	1	2	86
Misiones	10	9	190
Neuquén	7	133	58
Río Negro	8	15	204
Salta		46	62
San Luis			2
Santa Cruz		1	19
Santa Fe	117	8	10
Santiago del Estero		6	66
Tierra del Fuego, Antártida e Islas del Atlántico Sur	46	121	408
Tucumán	4	4	77
Catamarca	1		
Formosa		1	
Jujuy		2	

**Table 2.** Number of principal orders of edaphic fauna registred in the dataset.

**Phylum**	**Class**	**Order**	**No. registered**
Annelida	Clitellata		4
		Crassiclitellata	940
Arthropoda	Collembola		72
		Symphypleona	126
		Poduromorpha	333
		Neelipleona	12
		Entomobryomorpha	302
	Arachnida		58
		Trombidiformes	282
		Sarcoptiformes	2481
		Opilioacarida	5
		Mesostigmata	219
		Ixodida	4
